# Parental control and monitoring of young people's sexual behaviour in rural North-Western Tanzania: Implications for sexual and reproductive health interventions

**DOI:** 10.1186/1471-2458-11-106

**Published:** 2011-02-16

**Authors:** Joyce Wamoyi, Angela Fenwick, Mark Urassa, Basia Zaba, William Stones

**Affiliations:** 1 National Institute for Medical Research, P.O Box 1462, Mwanza, Tanzania; 2 University of Southampton, School of Medicine, Division of Medical Education, Boldrewood campus SO16 7PX, Southampton, UK; 3 London School of Hygiene and Tropical Medicine, Department of Epidemiology and Population Health 49-51 Bedford Square, London, UK; 4 Aga Khan University, Faculty of Health Sciences, 3rd Parklands Avenue, P.O Box 30270-00100, GPO Nairobi, Kenya

## Abstract

**Background:**

Parenting through control and monitoring has been found to have an effect on young people's sexual behaviour. There is a dearth of literature from sub-Saharan Africa on this subject. This paper examines parental control and monitoring and the implications of this on young people's sexual decision making in a rural setting in North-Western Tanzania.

**Methods:**

This study employed an ethnographic research design. Data collection involved 17 focus group discussions and 46 in-depth interviews conducted with young people aged 14-24 years and parents/carers of young people within this age-group. Thematic analysis was conducted with the aid of NVIVO 7 software.

**Results:**

Parents were motivated to control and monitor their children's behaviour for reasons such as social respectability and protecting them from undesirable sexual and reproductive health (SRH) outcomes. Parental control and monitoring varied by family structure, gender, schooling status, a young person's contribution to the economic running of the family and previous experience of a SRH outcome such as unplanned pregnancy. Children from single parent families reported that they received less control compared to those from both parent families. While a father's presence in the family seemed important in controlling the activities of young people, a mother's did not have a similar effect. Girls especially those still schooling received more supervision compared to boys. Young women who had already had unplanned pregnancy were not supervised as closely as those who hadn't. Parents employed various techniques to control and monitor their children's sexual activities.

**Conclusions:**

Despite parents making efforts to control and monitor their young people's sexual behaviour, they are faced with several challenges (e.g. little time spent with their children) which make it difficult for them to effectively monitor them. There is a need for interventions such as parenting skills building that might enable parents to improve their relationships with children. This would equip parents with the appropriate skills for positive guidance and monitoring of their children and avoid inappropriate parenting behaviour. As much as parents focus their attention on their school going daughters, there is a need to also remember the out-of-school young people as they are also vulnerable to adverse SRH outcomes.

## Background

Evidence has pointed to the influence of the family when dealing with a young person's behaviour and giving careful consideration to environmental factors and interpersonal relationships [1-3]. Family social characteristics have been found to influence the behaviour of children and their achievements throughout their life course [2-4]. For example, warm and supportive relationships have been found to provide young people with more security and confidence to meet academic challenges and to resist negative peer influences [5]. However, although home environments and parents in particular have a profound influence on the ability of the young people to develop in a healthy manner, studies indicate that young people also influence their parents' behaviour and play a role in generating the very social conditions that influence adverse developmental outcomes [6].

There is an increasing recognition of the fundamental influence of parent-child relationships on the sexual behaviour of young people. For example, appropriate parental care and assertiveness have been shown to have a positive role in reducing adolescent sexual activity [7,8]. Although much of the evidence on the influence of parent-child relationships on young people's sexual behaviour comes from studies conducted in developed countries [9-13], there is also a growing body of literature from sub-Saharan Africa (SSA) [14-17]. Several intervention programmes focusing on the role of parenting in improving adolescent sexual and reproductive health (SRH) have been implemented and experience from 30 of such programmes was described in a World Health Organization review (WHO) [18]. In East Africa, there have been efforts to explore parent-child relationships and specifically parent-child communication; for example, in Uganda Kinsman [19] and Muyinda [20] have studied the use of traditional forms of socialization such as the Senga, while programmes such as the "straight talk campaign" have demonstrated the general willingness of parents and other adults to create a supportive environment for young people [14]. In Kenya, programmes such as "families matter" work directly with parents and their children to improve intra-familial communication about sexuality and sexual risk [15]. In Tanzania, Nyalali et al. [17] have examined general parent-child relationships and pointed to the biases inherent in questionnaires with parents about their relationships with their children. Nonetheless, despite these efforts, there remains a lack of detailed knowledge and understanding of parental control and monitoring and how these relate to young people's sexual behaviour in the regional context.

A WHO review categorised relationships between parents and their children into five dimensions: 'connection', 'behaviour control (control and monitoring)', 'provision and protection', 'respect for individuality' and 'acting as role models' [21]. There is evidence mainly from the developed countries and especially the USA, that point to these dimensions being associated with health related outcomes. There is however, a dearth of literature from SSA on how parent-child relationships affect young people's health and on parental control and monitoring and why parents may be motivated to control and monitor their children's behaviour. Important questions remain as to the applicability of findings from studies conducted in developed countries to SSA. There is a need to understand the influence of parent-child interactions and control and monitoring on young people's lives and how this influence can be utilised or modified to benefit their sexual and reproductive outcomes. This paper explores the dimension of parent-child control and monitoring and how this influences young people's sexual behaviour particularly behaviours that would increase their vulnerability to HIV/AIDS (such as early sexual debut, having multiple partnerships and not using condoms and contraception) and unplanned pregnancy. Such understanding can be anticipated to support more effective and sustainable behaviour change interventions. In this study parental control refers to the rules and restrictions imposed by parents on their young people's activities and friendships, thereby controlling the amount of freedom young people have to do things without telling. Parental monitoring refers to situations when parents follow up or express concern about young people's activities and question their whereabouts.

## Methods

To understand parent-young person relationships and control and monitoring, the study emphasized the importance of interpretation as well as observation in understanding their behaviour [22]. Parents influence sexual behaviours through their expectations of young people. An understanding of this influence was gained through both observations of interactions within families and interviews and focus group discussions with parents and young people. Although the researchers' own interpretations were important, they were clearly delineated from those of the participants and, therefore, the researchers adhered as closely as possible to the participants' accounts as the basis for interpretation.

Ethical approval for the study was provided by the Tanzanian Medical Research Co-ordination Committee. Additional permission to conduct the study was granted at district, ward, and village levels (community and individual). In addition to seeking the consent of participants, for those aged below 18 years (the age of majority in Tanzania), consent was also sought from parents or caregivers. The purpose of, and methods for, the study were explained to potential participants, who provided verbal consent prior to participating.

### Design

This study employed an ethnographic research design. Data were collected using participant observation (PO), in-depth interviews (IDIs), and focus group discussions (FGDs). A combination of PO, IDIs and FGDs increased the understanding of complex issues related to young people's sexual behaviour, and provided a detailed understanding of parental control and monitoring as understood and practised in the study. PO provided background information on the interactions in families and parent-child relationships and allowed for the understanding of the social realities of the participants by experiencing the circumstances in which they live. FGDs provided an understanding of attitudes about parent-child interactions at the wider community level and IDIs explored parent-child interactions at a more personal level.

### Study setting and participants

The study was conducted in the Kisesa demographic surveillance site in North-Western Tanzania. The predominant ethnic group was Sukuma. The main religion was Christianity, while the principal economic activity was farming. The study participants were young people aged 14-24 years old and parents/caregivers of young people of that age. Out of the 46 IDIs, 25 were conducted with women (14 young women, 11 with female parents/caregivers) and 21 with men (12 young men, 9 male parents/caregivers). Eight of the FGDs were conducted with women (5 with young women, 3 female parents/caregivers) and 9 with men (6 young men, 3 male parent/caregivers). Most of the young people were either attending or had completed primary school. A very small number were attending secondary school. Some of the participants in their twenties were still in school. It should be noted that in the Tanzanian education system classes are repeated, sometimes resulting in students still being in primary classes into their twenties.

Both male and female parents were included as participants as we were interested in understanding the interactions between parents and young people from both perspectives and because we were interested in looking at parents' role in potential SRH interventions as they are the main socialization agents in families.

### Data generation procedure

Data were collected in 2007 by a female graduate researcher (first author) and a male research assistant, a sixth form secondary school graduate. PO involved spending eight weeks in one village, forming friendships with young people and their families and observing interactions between young people and their parents. The two researchers resided with two separate families but interacted with other families in the village. A total of 20 families (15 both parent, 5 single parent) were visited several times over the eight weeks. The observations were conducted with the aid of a checklist to ensure that they were focused on the objectives of the study. Jottings were taken in the course of the day and detailed notes were prepared at the end of each day describing important observations for the day.

At the end of PO, FGDs and IDIs were conducted with some of the participants from the PO village and from six other villages. A snowballing approach was adopted for the selection of participants for FGDs and this ensured that all the participants knew each other well and were free to discuss sensitive issues in each other's presence. Data were collected in two phases. During the first phase of the FGDs, three days were spent getting to know and recruiting pre-existing friendship groups [23]. The FGDs focused on broader issues related to parenting and young people's sexual behaviour. The FGDs with parents were organized according to gender, while those with young people were by gender and schooling status (in and out-of-school). The selection of participants for the second, follow-up phase of FGDs was based on a theoretical sampling approach [24]: two more FGDs, each with young women and men were conducted to explore further issues that had emerged from preliminary in-depth interview analysis.

Participants for IDIs were selected from FGD participants through purposive sampling (schooling status, sex, responses given during group discussion). The IDIs were held with FGD participants so as to build on the rapport built during the group discussion and to explore at a personal level some of the issues that had emerged in the FGDs. Initially, 39 interviews were conducted. After reviewing data generated in the initial 39 interviews, decisions about whom to interview were informed by the preliminary analysis and emerging explanations from the initial data. Theoretical sampling was used for the selection of participants for seven follow up interviews: three parents were interviewed a second time to elicit a more detailed understanding of some of the issues they had raised and a further 3 interviews were conducted with new participants to explore issues that had emerged in the first interview phase.

Data were collected on family socio-economic status, family material and social support and young people's sexual behaviour. For this paper, we specifically asked about: parent-child control and monitoring strategies, gender differences in control and monitoring, parental presence and young person's control and monitoring, decision making dynamics in families and interactions within families.

### Analysis

The data were transcribed, translated into English, entered into QSR NVIVO 7 software, and coded. A pragmatic approach to analysis was adopted using a combination of an already designed coding scheme (anticipated codes) and grounded codes. Grounded codes were developed through a thorough reading of the data and reflected the language and ways of expressing ideas portrayed by the participants. The anticipated codes were developed from the research objectives, prior knowledge, and repeated reading through of the data during the early stages of the analysis and these were then refined in the light of further data. Thereafter, codes were developed into more conceptual categories and, finally, themes. In the presentation of PO findings, pseudonyms have been used in the examples.

## Results

### Context of parent-child relationships and Characteristics of control and monitoring in families

Generally, parent-child interactions were structured along gender lines and young people were expected to show respect for all adults. We have previously commented on these interactions: for example parents often showed a lack respect for their children's individuality and, most particularly, towards female children [25]; what communication there was about sexual health was mainly along gender lines specific [26]. Furthermore, most parents were not able to provide adequately for their children's material needs and so young people had to make provision for themselves [25,27]. Many young people reported that their parents were poor role models partly because of their own sexual behaviour (e.g. having multiple partners) but also because of their inability to provide adequately for their needs whilst at the same time spent money unwisely (e.g. on alcohol) [25].

Although parents were not directly asked about ways they controlled and monitored their children in this study, the issue repeatedly came up when discussing some of the things that triggered a discussion about SRH with their children. Parental control of young people in this setting comprised of activities such as: setting limits on young people's activities (i.e. time spend outside home, appropriate dressing and ensuring young people adhered to values on expressing respect) and friendships. Parental monitoring involved: following up on young people's whereabouts; enquiring about anything they saw their young person with (e.g. clothing, money); and following up on young people's friendships.

### Motivations for parental control and monitoring

Generally, parents were motivated to control young people's sexual behaviour for reasons such as: fear of HIV and sexually transmitted infection (STI) infection, unplanned pregnancies, benefits from bride wealth, and to ensure the good social reputation of their families. Moreover, unmarried pregnancy was perceived as a sign of failure on a parent's part; unmarried young women with children were referred to using a derogatory term of *msimbe *(singular) and *wasimbe *(plural). As they had children and were not married, *wasimbe *women were viewed as less attractive for marriage and, furthermore, were a burden to their parents since most of them were unemployed. Many parents clearly did not want their daughters to be classed as *wasimbe *due to this economic burden, but most importantly, because it would lower the social reputation of the family.

Marriage for daughters remains the preferred goal for most parents and this provided the motivation for them to monitor their daughters' sexual behaviour. It is, however, noteworthy to mention that in some rare cases, the primary aim was to ensure that daughters were educated and families reminded young women about the value of education and the incompatibility of education and premarital sex.

There was a feeling among most people that, it was useless for parents to strictly control and monitor young women who were already sexually active and the *wasimbe *because they were perceived as sexually experienced and could not easily be restrained from sex. A female parent talked about the reputation of *wasimbe *as follows:

*They can't follow up a msimbe because she has left school, she has a child, you can't get bride wealth. A school girl and the unmarried woman without a child are followed up most *[IDI, 37 year old married woman].

Parents described a girl being able to complete primary school without having had unplanned pregnancy as 'luck' and this also required lots of parental effort through control and monitoring:

*A child completing standard seven without pregnancy depends on the parents. For you to bring up your family in a good way, you have to keep monitoring your child so much so that she doesn't leave home, because if she goes there[social places] she will discuss many things [sex] with friends *[IDI, 35 year old married woman].

### The role of time in control and monitoring

The families' economic activity were very important in determining the amount of time parents spent with their children which was important for any effective monitoring to occur. In some families parents were absent for the whole day; for example working at the rice mill or stone quarry, undertaking petty trade involving taking farm produce to the city.

Family structure seems an important factor affecting both the level of parental control and monitoring and the sexual decisions of young people. In contrast to both parent families where one parent was likely to be around when the other was away, single parents tended to undertake very little monitoring. The priority for single mothers was usually to work hard in order to provide the basic needs for their family and they rarely had time to follow up on what their children were doing. Those who engaged in labour that involved a whole day of heavy physical work were referred to as *walala hoi *(a person who sleeps exhausted from doing heavy manual work). *Walala hoi *mothers left home early in the morning and returned late. Young women from such families reported that when their mothers returned home, they were so tired they hardly had any conversations together. The following is an illustration from participant observation:

*Paula said that their mother usually leaves home at 7.00 a.m to go to work at the rice mill. She comes back home at 7.00 pm and this depends on the season of the year and availability of work and therefore, she may come back even later. Yesterday their mother came back home at 8.30 pm and by the time she arrived the family had already had dinner and the children were already asleep *[PO notes, conversation with a16 year old girl out-of-school woman].

Spending time with daughters, however, did not necessarily indicate an increased level of control and monitoring. Some mothers in both parent families also did not follow up on their children's sexual activities. Although they spent more time with them, we observed that they did not question their daughter's whereabouts, whilst male family members who often spent less time with their children did. Male parents reported that since mothers spent more time with their daughters they should know what their daughters did and what they were able to afford. Male parents reported that in the past, mother-daughter relationships were much closer than nowadays and in some cases accused their wives of collaborating with their daughters in the planning of sexual activities. When a girl was discovered to have an unplanned pregnancy, the mother would be questioned because it was assumed that she had been aware of her daughter's sexual activity but had not informed the father. The following is an excerpt from a discussion with a male participant:

*Mother knows it because, if she hasn't given her money and she sees her with money and she doesn't ask...even the girl knows that her mother knows *[IDI, 35 year old woman, key informant].

### Gender differences in monitoring young people's sexual behaviour: *"Boys are not asked because it is our tradition"*

Generally, parental monitoring of sons' sexual activities was minimal. Some fathers said that they knew when their sons had gone out to look for women and were not bothered by this as long as their sons had completed their tasks at home. Parents' toleration of their son's sexual activities was partly because of the traditional social norms surrounding male power and sexual behaviour but also because parents believed that sons would not inflict the economic burden of an unplanned pregnancy.

Young women reported that, unlike their brothers, they were not free to do what they wanted. In a group discussion with out-of-school young women, they said that most parents did not ask their sons about where they had been because it was part of their culture for boys to be free:

R1: The boys are not asked because it is our tradition. A male child is not followed up in families

*R2: You cannot follow up a male child because he cannot bring a loss, but this female child can bring a loss. The loss is about pregnancy. She can get a boyfriend out there and become pregnant. Now it is mostly the parent who will struggle *[FGD, out-of-school young women].

A young woman talked about her experience as follows:

*My brothers can sleep out...father asks, 'where is so and so', he is told and he doesn't say anything...but when I spend a night out, immediately he arrives he asks, 'where is so and so, where did she sleep' *[IDI, 20 year old woman].

Some parents were against sharing a sleeping house with their adolescent children due to cultural norms prohibiting this. There were however, a few who reported that they ignored the cultural norms and shared a house with their daughters at night for ease of monitoring them. A male parent said that his sons could do whatever they wanted because they cannot become pregnant:

*I sleep with my daughters in the same house so that I can prevent them from becoming pregnant. This is because if they sleep in a separate house on their own they can bring boys. But for the male children, even if they make a girl pregnant, we can refuse it, but for the girls if they get pregnant I have to bear the burden here at my home because you cannot refuse the pregnancy and she is carrying it. It will also force me to start the work of looking for whoever made her pregnant *[PO notes, a conversation with a male parent].

The role of mothers in their sons' sexual decision making was twofold: permitting or restricting. Mothers in some families especially the single parent ones seemed to value their sons more than their daughters and were usually not restrictive on their activities. Mothers reported that in addition to their sons playing a providing role, having a son also enhanced their social respectability. Most of the single mothers were more permissive towards their sons' sexual activities than they were in their daughters. They indicated that they respected them and did not question their activities because they thought it was not proper; they in fact also used them to monitor their sisters' sexual activities:

*She [daughter] feared him [younger brother] than me...She fears her younger brother because he is a man...when he is at home she returns early...Also, her brother questions her for chatting with men [potential sexual partners] *[IDI, 35 year old single mother].

Importantly, there were a few parents who said that they did sometimes follow up on their sons' whereabouts and attempted to prevent their sons' engaging in premarital sexual activities because of the fear of HIV/AIDS and because of the economic and social consequences (i.e. imprisonment) of making a school girl pregnant. Young men did sometimes end sexual relationships with their girlfriends when they realised that the girls were still in school, however, most reported that they kept their sexual relationships secret because they feared being forced to marry a woman they did not love just because they were having a sexual relationship with her. For example:

*She (girlfriend) had come to visit her brother. Then we started the relationship, but when I realised she was a pupil, I stopped because once you make her pregnant, it really becomes a loss...because first of all you may risk your life, you may be imprisoned for many years or if you run away, then, your parents may be fined *[IDI, 20 year old man].

As indicated in this excerpt, young men who understood the consequences of having sexual relationships with school girls avoided them as sexual partners. It is interesting to note that although some of the out-of-school young men have become careful about having sexual relationships with school girls, the girls themselves sometimes appeared willing to have sex with them, even concealing their schooling status; this could be attributed to the potential financial benefits to be gained.

#### Mothers' monitoring techniques for daughters

Mothers who were keen on restricting their daughters' sexual activity appeared to have adopted several ways to monitor their daughters' behaviour. Examples included: verbal warnings; use of trickery (faking stories about their daughter so as to entice them to talk about their sexual activities); use of other family members and relatives as informers; and physical examination of their daughters' private parts. The commonest monitoring technique reported was the use of others (relatives and other family members) to inform them of their daughters' sexual behaviour when they themselves were away from home.

A few mothers were convinced that they were bringing up their daughters well because they had not had unplanned pregnancies like some other girls from the village. They were selective in the places that they allowed them to go and relied on others as informers:

*...I allow them to go to church because they go with their brothers and when they finish singing, they can move around but they have a limit. They don't just go anyhow. Now if any of them talks with men, the other siblings will inform me *[IDI, 54 year old married mother].

Some mothers reported their daughters' behaviour to their husbands, telling them when they thought their daughters had started sex or were engaging in behaviours that might lead to it. One of the ways they followed up on their sexual activities was by asking them to account for unexpected items (clothes, shoes). If she mentioned that she had been given something by a relative, the mother then followed this up with the relative to establish the truth:

*Perhaps you see that child with money, you ask 'where did you get it from?'...that can make you know that this child has begun sex *[FGD, female parents].

Their aim was to try to prevent their daughters taking money from men that would indicate willingness to engage in sex.

A few young women reported that their parents applied physical discipline through beatings whenever they suspected them of engaging in sexual activity and this could occur even for single parents (after pregnancy). A 20 year old *msimbe *woman who had been beaten for taking money from a man talked about her experience as follows:

*She beats me when I take money from men, she tells me that I shouldn't accept that money...she also beats me when I come home late...She says I was late because I was with a boyfriend [*IDI, 20 year old *msimbe*].

This parent's monitoring techniques seemed to have worked for her daughter because she mentioned that when she got married at the age of 16, she was a virgin. She was instructed about how to have sex by the same mother and grandmother. She also said that when the man who had married her approached her for sex (before marriage), she told him to come and talk to her mother about it. That is when the man came with a marriage proposal and married her through *bukwilima *(traditional marriage ceremony). Unfortunately the marriage lasted for less than a year. Young women's marriages not lasting for long could be attributed to the lack of opportunities for them to know their spouse properly before marriage. Some of the young women acted on parental instruction that if a man seduced them they should tell him to come and see their parents.

One mother talked about how she used the technique of physically inspecting her 15 year old primary school daughter's private parts so as to ensure that she remained a virgin. In order to facilitate her inspection, she used scare tactics such as telling her daughter that if she slept with clothes on, she would not be able to give birth: she wanted her daughter to sleep without underwear so that she could easily inspect her private parts:

*My neighbour told me, 'this woman will spoil your daughter because there is a certain boy who meets her at her home [woman's]...Apart from that I always have a tendency of sparing some time during the night when she is sleeping ...I usually go to check in her private parts. Since last year[daughter was 14 years old] I have been doing that work...I always check to see if she has already slept with a man... it can be seen if someone has broken her virginity...I usually touch in her private parts and if she turns around I tell her 'sleep well, cover yourself' ...you had slept naked. Then I pretend to cover her to conceal my intention and yet inside my head I know what I am after *[IDI,34 year old mother].

This monitoring techniques portrayed the mother's lack of confidence in her daughter's behaviour and as noted in other work [21] a lack of respect for her privacy. This mother thought that by touching in her private parts she could know if her daughter had engaged in sex and thus find a reason for reinforcing her advice about the consequences of premarital sex. Unfortunately, by the time she thought it was the right time for her to communicate prevention messages, her daughter had already had sex.

Another monitoring technique applied by a few mothers on their primary school daughters was the use of trickery. Some reported that they discouraged their daughters from having sex by making up a story that they had seen them with men. This was used as a way to get daughters to talk about themselves and hopefully about their partners if they had any. However, this technique seemed to close down communication rather than open it up. A mother who had used this technique and thought it worked for her said:

*It is a trick, because I just formulate things that I have not seen. I start telling her, 'I saw you there, do you want to deny?'... 'Now do you want me to bring him [man] here? ...or should I tell you to bring him'...She tells me, 'mother you know, I was asking him to pay me my debt'... then I tell her, don't do it again *[IDI, 35 year old mother].

Due to lack of confidence in her daughter's behaviour, the mother in the above excerpt used tricks to manipulate her daughter to talk about something that she predicted was going on. Since school girls knew they were expected to abstain, they would not have talked about their sexual activity with their mothers and hence the mother's use of trickery. Therefore, mothers' use of trickery to get daughters to talk about their sexual relationships is a contradiction to the societal expectations on secrecy in sexual relationships [28]. As much as mothers may not talk about sex with their daughters at the right time, it is clear that they are interested in knowing when they became sexually active. When daughters were away from home, they suspected them for engaging in sex as reflected in their use of tricks to get them to talk about something they (mothers) imagined.

Some of the other monitoring techniques employed by mothers such as hanging around their daughters' sleeping place at night were clearly not effective in preventing their daughter's from engaging in sex. This confirms what some of the parents and young people had mentioned about the most effective control of young people's sexual activities as being the young people themselves since it was not possible for the parents to be with their children everywhere. Two young women now married talked about how their mother had thought that she was monitoring them but in reality they were able to do what they wanted: they had been cleverer than their mother because despite her close supervision, they still managed to sneak out at night to meet their sexual partners:

*Eva (26 year old married woman) slept in a separate house from her mother. She shared this with her elder sister. Every evening their mother used to stay in the house where they slept chatting with them until 10.00 pm. She could then wake them up at 5.00 am. But despite of their mother being with them most of the time they still managed to sneak out. She used to sneak out at 11.00 pm and come back at 4.00 am. She put it as, 'We had very complicated/hard science [cleverer/cunning]. Mother could never be able to see us. She wondered how both of us had unplanned pregnancy *[PO notes].

Parental control and monitoring of young people's sexual activities was important in the decisions young people made concerning sex. However, as much as some parents thought that strict control was helpful, it was observed that strict monitoring encouraged deceit among young women to avoid conflict with their parents. Young women reported that they had developed tactics to avoid conflict with their parents. For example, some said had decided to engage in petty trade so that they could easily account for the gifts and money they received from their sexual partners. The following is an excerpt from participant observation during a conversation with a 14 year old standard 6 girl living with both parents:

*Maria said that she sometimes uses the money given to her by her 26 year old boyfriend to buy clothes. She is never asked by her parents because they know that she hawks vegetables *[PO notes].

Some parents reported that parents being very strict only resulted in frustration and shame when their daughter ended up with unplanned pregnancy. When asked about his thoughts on whether strict parental control would prevent a child from engaging in sex, one of the parents said:

*I mean...You know the body ...even if he is very harsh, it is impossible to prevent a child... There are examples of very harsh parents who monitored their daughters everywhere, but the end results were the children getting pregnant *[IDI, 42 year old father].

#### "If a father is there, children fear": Father's presence in the family

Whether or not a fathers' physical presence had an impact on young women and men's sexual decision making was debatable: some felt it made a difference, others not. On the one hand, young women from families with fathers often received stricter control compared with those living with their mothers only; on the other hand daughters from families with a father had to be more secretive as they continued to engage in sex. A mother talked about this in the following:

*There is no difference, concerning the issue of pregnancy by family type. I can say although a girl with a male parent can be tightly controlled, she will use all her opportunities. So the issue of unplanned pregnancy can happen in any family *[IDI, 35 year old married woman].

Those who believed that a father's physical presence made a difference argued that this worked through instilling fear:

*If a father is there, children fear. She is sent on errands but she fears to do it [have sex]. Even if she gets it [pregnant], she will make efforts to abort so that they can't know it at home. But if she lives with mother alone, and mother is a very quiet person, if she is asked 'why are you like this [pregnant], she just keeps quiet *[IDI, 35 year old mother].

*You know in families headed by fathers, daughters sometimes fear. So it is not easy for her to engage in sex ...she guards herself very much....She fears that if father knows about this issue, he may quarrel me. But in homes with mothers, even if she knows it, she understands that this is a normal thing because even herself [mother] is single *[IDI, 42 year old father].

It is interesting to note that most of the participants who strongly felt that a father's physical presence was important came from single mother families, either the single mothers themselves or young people from such families, who believed that fathers could be more strict:

*For example, if you find that their father is very stern, there are very few who get pregnant *[IDI, 38 year old woman].

Fathers also played a protective role when their daughters were harassed by men who seduced them, instilling fear in these men. This view confirmed what young men reported about fearing to have sex with girls from families reputed to have strict fathers. This may mean that girls with such fathers may have been more protected than those from single mother families or those with less strict or less caring fathers. One of the two primary school girls talked about her experiences in the following:

*One day I was at the weekly market, a certain man gave me money but when I refused he started beating me. I came and told father because he really hurt me. Father made a follow up on him and when he got him he told him 'you have injured my child, now are you seducing her by force? ...you should never repeat that' *[IDI, 15 year old girl].

Similarly a mother talked about the way her daughter reported the harassment she got from a man (potential sexual partner):

*She said "Father, so and so came and hugged me and touched my breasts"... fortunately she had hardly finished talking when that boy passed [along the road]. He was warned and he never repeated again... he feared and ever since that time I have never heard any complaints of her being seduced or treated unfairly by men *[IDI, 34 year old married woman].

### Parental control and risk

Most of the parents were aware of the places and times that carried the highest risks for sexual activity; examples of these were social events such as discos and video shows and celebrations such as New Year, Easter and Christmas. Discos and video shows were believed to encourage sex through the arousal of sexual desire from viewing pornographic movies and the dancing styles that were deemed sexualised. They were also perceived as ungodly and associated with HIV risk:

*I talked to them about protecting themselves and about walking at night, perhaps to go to the disco and such things...things that stimulate body lusts...Ee, the disco stimulates body lusts because the disco is conducted at night, you find that the clothes they put on are unpleasant ...they dance half naked *[IDI, 50 year old father].

Even though parents attempted to stop their children from going to video shows or discos, some young people still went to these places secretly. Some male participants, who attended discos, said they would never allow their sisters to go to such events. They, however, mentioned that when they attended, they expected to dance with girls. They perceived such girls as promiscuous and spoiled 'beyond repair' (*walioshindikana*) and only fit for such entertainment but not long term relationships. In some instances, a girl found attending discos would result in her being expelled accompanied by serious threats:

*X said that there was a time when Neema was performing in the disco in their village. When Neema's father heard about this, he said that she should not return home and that if she came, he would kill her. Neema feared this threat and decided not to return home but went to the lakeshore fishing community. Since then [a year] they did not hear about her until today when she saw her at the dispensary. Neema had come back home to her parents after falling ill. X said that she thinks that she is infected with HIV *[PO notes].

The above example is a clear indication of the fears that young people may have concerning parental punishment for socially disapproved behaviour. Leaving home also might mean additional exposure to risky activity as most go to live with friends and relatives who are less strict. For instance, the lake shore fishing communities like the one where Neema went to live were reputed to be 'high risk': they are full of young people, most of whom have run away from home due to strict parental control and other disagreements.

Parents acknowledged that it was difficult for them to have total control over daughters because they sometimes leave home alone to go to church, market, fetch water, or visit relatives among other activities. They said that girls are approached by men for sex when they are on errands. Therefore, some of the participant's views about no one being able to successfully control young people's sexual activities other than the young people themselves seemed realistic. This is because of the secretive and opportunistic nature of most sexual encounters.

## Discussion

The findings demonstrate that parents may be motivated to control and monitor their children's sexual behaviour because of the immediate and future economic and social benefits. The immediate benefits were those related to social reputation as a result of their children's behaviour. The long term benefits relate to ensuring marriage and benefiting from bride wealth if their daughters were reputed to have 'good behaviour' and this has been reported previously [28]. Bride wealth involves significant transfer of wealth from the man's family to the woman's and the amount of bride wealth exchanges are partly determined by a woman's sexual reputation.

Parental control and monitoring varied with family type, schooling status of the young person and the child's gender. It was also linked to the ability of the family/parent to provide for material needs for their children. The gender differences in parental control could be attributed to patriarchal conditions that socialised male children differently from the girls and expected them to be aggressive in seeking girlfriends while girls were not [25]. The focus on girls regardless of schooling status was also because they wanted to prevent them from undesirable SRH outcomes such as unplanned pregnancy and HIV.

There were varied opportunities for control and monitoring in different family types and parental presence seemed important. Young people from the single parent families were less controlled regardless of their schooling status. As noted in Uganda, young people reported that their sexual activities happened when parents were away or when they were staying with other relatives [8]. The interpretation of parental presence as a restraint observed in this study is consistent to that reported elsewhere [8,29]. However, the effect of fathers' presence on young people's undesirable SRH outcomes (such as early sexual debut, unplanned pregnancy) observed elsewhere [29-31], was not consistent with what was found in this study. The frequency of unplanned pregnancy did not seem to differ with a father's presence in the family. What differed most among the young women from the single mother and those from both parent families was the way they handled their sexual relationship, with girls living with fathers being more secretive than those living with mothers alone.

The findings show that parents tend to monitor their daughters' sexual activities more than their sons. They employed a variety of techniques to monitor their daughters' sexual behaviour. Some techniques involved physical inspection of their daughter's private parts and the use of tricks to get daughters to talk about their sexual activities. These were invasive and intrusive and can be seen as countering basic rights of their children (see for example, the WHO review [21]).

Studies conducted in developed countries have noted 'lower level' parenting techniques such as following up on daughters' whereabouts and monitoring how they spend their money to control early sexual activity, number of sexual partners and condom use [32]. The invasive techniques outlined in this study have not been documented before and it is important to note that meanings, motivations and techniques associated with parental control and monitoring may vary across cultural contexts.

Fathers' protection for daughters could be classified into direct and indirect. The direct protection could be related to the direct communication about abstinence and the follow up on their activities so that they could not get into situations that would lead to sexual activity. The indirect protection could be the one related to their characteristics (i.e. harsh, lenient) and the way other men (daughters' potential sexual partners) perceived them and as a result decided to or not to seduce their daughters. Fathers' efforts in the control and monitoring of their children's sexual activities would however, need to be combined with those of other adult family members for them to have a more meaningful impact.

Apart from parents having different behavioural expectations for their children by gender, these also varied with the child's schooling status. The parental belief that schooling and sexual activity were not compatible because unplanned pregnancy among girls could lead to school drop-out, meant that they mainly focused their attention on the prevention of sexual activity among young women still in school. The findings about the expectation of abstinence for school girls as one of the fundamental sexual norms is consistent with what was observed in another study in rural Tanzania [28]. Parents tried to provide for their in-school daughters' material needs, controlled and monitored their activities and frequently communicated about avoidance of premarital sex. Parental focus on the in-school young people and rarely on the out-of-school has also been reported in other studies [33,34]. In line with the parental beliefs about sexual activity and education not being compatible [28], the idea of encouraging abstinence among schooling young people may be helpful in protecting them from HIV/AIDS, but only if it is possible for them to adhere to it. However, this offers an insight into a useful method of health promotion: encouraging the early start of schooling, keeping young people in education (primary and secondary) and avoiding school drop-outs. Such an effort may be more acceptable to parents as it is already in line with their beliefs. Moreover, if young people remain in education until they complete their schooling they are more likely to be exposed to increased SRH information which may be useful in promoting their sexual health. On the flipside, parents lack of focus on supporting and monitoring out-of school young people means that this group (both genders) is at increased risk of HIV/AIDS and other undesirable SRH problems. Several studies have also pointed at this group as being at increased risk [33,34]. As indicated in the findings in this study, the expectations that out-of-school young people should fend for their needs and sometimes that of their families, puts them (particularly women) at risk of engaging in transactional sex to meet them [27]. Parents and other SRH interventions focusing their attention mainly on in-school young people and mainly ignoring those out-of-school does not seem very helpful in preventing young people from undesirable SRH outcomes such as STIs including HIV and unplanned pregnancy.

As much as we agree with authors who advocate that SRH education should start before sexual debut for it to have a more desirable impact on sexual behaviour and hence their focus on the young people in school [35,36], but parental as well as external SRH interventions focusing on the out-of-school young people is also essential. This is because they may influence the sexual decision making of those still schooling given that some of the sexual partners of the school girls are out-of school men. Moreover, the gender relations and traditional beliefs around female subordination may not make it easy for a school girl who has information on protection use to insist on their use when she is with a sexual partner who is unaware of condoms or reluctant to use them. Interventions may achieve much in terms of the sexual health of the school going young people, if they too are focused on the out-of school young people, especially men, who seem to be playing a major role in sexual decision making. Hence, this study's focus on both the in and out-of-school young people, has contributed to the understanding of the different contexts in parent-child relationships, particularly control and monitoring and how parents pro-sexual and abstinence messages and control and monitoring measures varied.

This study's utilization of participant observation in addition to interviews and focus group discussions has been very useful in providing a detailed understanding of parental control and monitoring in a rural setting. As noted by others [17,37], self-reported data can be problematic due to strong social desirability bias. The use of participant observation allowed for the observation of interactions within family members and between parents and their children and hence provided some unprompted information on the subject. Notwithstanding, the study's focus on one rural setting means that the findings may not be easily applicable to urban settings where there might be big differences in socio-economic status and parent-child relationships. Further research utilising quantitative approaches would be useful in establishing the extent and impact of the different monitoring techniques on young people's sexual behaviour and health outcomes.

## Conclusions

Parents are making many efforts to prevent their young people from SRH risks. They are however faced by several challenges which made it difficult for them to effectively control and monitor their children. For example, economic constrains made it difficult for them to spend time with their children and inability to provide also seems to have an effect on their parental authority. Some of the monitoring techniques employed by parents may be unethical and point to the need for them to be provided with support and help with developing skills which enable them to remain engaged in their children's activities and respect their individuality whilst avoiding conflict.

As much as parents focus their control and monitoring on their schooling children, it is desirable that they have a role to play with out-of-school young people as they are also at increased risk of HIV, STIs and unplanned pregnancy.

## Competing interests

The authors declare that they have no competing interests.

## Authors' contributions

JW conceived and designed the study, coordinated and conducted field work and analysis, and wrote the first draft of the paper. AF provided technical advice on the study design, analysis and interpretation of data and write-up of the paper. MU helped to coordinate the field activities and provided feedback on the draft paper. BZ provided technical advice and contributed to the write up. WS participated in the overall design of the study, provided technical support on the data collection, analysis and write up of the manuscript. All authors read and approved the final manuscript

## Pre-publication history

The pre-publication history for this paper can be accessed here:

http://www.biomedcentral.com/1471-2458/11/106/prepub
